# The Long-term Effect of Inferior Turbinate Surgery Techniques on Nasal Obstruction and Quality of Life

**DOI:** 10.1177/00034894211049573

**Published:** 2021-10-06

**Authors:** Teemu Harju, Jura Numminen

**Affiliations:** 1 Department of Otorhinolaryngology, Tampere University Hospital, Tampere, Pirkanmaa, Finland

**Keywords:** clinical outcomes research, miscellaneous, nasal and sinus surgery, microdebrider, quality of life, visual analog scale, nasal obstruction, rhinology, otolaryngology, nasal turbinate, radiofrequency ablation

## Abstract

**Objective:**

The aim of the study was to compare the long-term effects of radiofrequency ablation (RFA), microdebrider-assisted inferior turbinoplasty (MAIT), and diode laser techniques on the severity of nasal obstruction and quality of life (QOL) in a 3-year follow-up.

**Methods:**

The patients filled a Visual Analog Scale (VAS) regarding the severity of nasal obstruction and the Glasgow Health Status Inventory (GHSI) questionnaire preoperatively and during the control visits at 3 months and 3 years. Acoustic rhinometry was also performed. A total of 78 patients attended both control visits.

**Results:**

All 3 techniques improved the VAS score for the severity of nasal obstruction and the GHSI total score significantly compared to the preoperative values at both 3 months and 3 years. Compared to the preoperative values, all 3 techniques increased the V2 to 5 cm values significantly at 3 months. After 3 years, compared to the preoperative values, the MAIT (*P* = .005) and diode laser (*P* < .001) still had a statistically significant volume increase in V2 to 5 cm, whereas the RFA (*P* = .06) did not achieve a statistically significant effect.

**Conclusion:**

The RFA, MAIT, and diode laser all improved both the patients’ subjective sensation of the severity of nasal obstruction and QOL significantly. The response was sustained during the 3-year follow-up period with all 3 techniques. A weakening in the objective treatment response to RFA was found in the longer follow-up, but that did not cause a weakening of the patients’ subjective treatment response.

## Introduction

Chronic nasal obstruction is a common nasal symptom that has many adverse effects, including mouth breathing, dryness of the oropharynx, nasal speech, disordered sleep, restlessness, malaise, reduced lung volume, and overall reduced quality of life (QOL). In chronic rhinitis, the long-standing swelling of the submucosal tissue may become irreversible, leading to inferior turbinate enlargement, which is one of the main causes of chronic nasal obstruction.^1^ Conservative treatment of inferior turbinate enlargement is based on intranasal corticosteroids.^2^ If the topical treatment fails, inferior turbinate surgery can be considered.^3,4^

In recent decades, inferior turbinate procedures have concentrated on aiming at the reduction of the turbinate, with an improvement in nasal obstruction while maintaining nasal function and minimizing complications.^5^ In most of the cases, surgical treatment is targeted at the medial and inferior submucosal layer of the inferior turbinate.^6^ Various surgical techniques have been described for the reduction of enlarged inferior turbinates. The mucosal-sparing techniques of microdebrider-assisted inferior turbinoplasty (MAIT) and radiofrequency ablation (RFA) are commonly used worldwide.^5^ Of the several available laser techniques, diode laser treatment has also gained popularity.^7^

The final conclusion on the efficiency of inferior turbinate surgery should be based on the long-term effect of the treatments on the severity of nasal obstruction and the QOL. There are previous reports on the RFA, MAIT, and diode laser techniques showing the long-term efficacy in the treatment of chronic nasal obstruction.^8-10^ However, there are few studies comparing the effects of various techniques on the severity of nasal obstruction and QOL with a follow-up of several years.

The aim of this prospective randomized study was to compare the long-term effects of 3 common inferior turbinate surgery techniques—RFA, MAIT, and diode laser—on the severity of nasal obstruction and the patients’ QOL in a 3-year follow-up.

## Methods

This prospective randomized study was carried out at Tampere University Hospital, Tampere, Finland, between February 2014 and June 2020. The institutional review board approved the study design (R13144) and all patients provided written, informed consent.

### Patients

A total of 98 consecutive adult patients with enlarged inferior turbinates due to persistent year-round rhinitis were enrolled in this study. The patients presented symptoms of bilateral nasal obstruction that had not responded to a 3-month trial of appropriate treatment with intranasal corticosteroids. Patients with significant nasal septum deviation affecting the nasal valve region, internal/external valve collapse/stenosis, chronic rhinosinusitis with or without polyposis, previous nasal surgery, sinonasal tumor, severe systemic disorder, severe obesity, or malignancy were excluded.

Cone beam computed tomography (Planmeca Max, Planmeca, Helsinki, Finland) was used to exclude patients with chronic rhinosinusitis from the study. Serum-specific IgE-level measurements were used to identify patients with an allergic sensitization. Allergic sensitization was defined as a specific IgE > 0.35 for any common airborne allergen (cat, dog, horse, birch, grass, mugwort, D. pteronyssinus, and molds).

The definition of inferior turbinate enlargement was based on persistent bilateral symptoms, a finding of bilateral swelling of the inferior turbinate in nasal endoscopy, and the evident shrinking of both turbinates in a decongestion test. The nasal response to the topical vasoconstrictor 0.5% xylometazoline hydrochloride (Nasolin, Orion, Finland) in both nasal cavities 15 minutes before obtaining the second measurement was evaluated objectively using acoustic rhinometry (Acoustic rhinometer A1, GM instruments Ltd, Kilwinning, UK). An improvement of less than 30% in anterior nasal cavity volume (V2-5 cm) in one or both nasal cavities was considered normal and those patients were excluded from the study. The limit value of 30% was chosen based on previous literature.^11-13^

### Randomization and Groups

Patients were consecutively randomized into placebo, RFA, MAIT, and diode laser groups at a ratio of 1:2:2:2 using Minim, an MS-DOS program that randomizes patients to treatment groups by the method of minimization. Proportional amounts of patients with allergic sensitization were kept similar for each group. Age and sex distributions were also kept similar for each group.

### Surgery

The surgical treatment was performed in similar circumstances at the day surgery department of the hospital’s ENT clinic. All surgical procedures were performed by the same surgeon (TH). The procedures were carried out under local anesthesia with the patient’s eyes covered. First, the inferior turbinate was topically anesthetized using cotton strips with a mixture of lidocaine 40 mg/ml (Lidocain, Orion, Finland) and 2 to 3 drops of epinephrine 0.1% in 5 to 10 ml of lidocaine. Some 1.5 ml of local anesthetic (Lidocain 10 mg/ml c. adrenaline 10 µg/ml, Orion, Finland) was then applied to the medial portions of both inferior turbinates. All the procedures were performed under the direct vision of a straight, 4 mm-diameter, 0° endoscope (Karl Storz, Germany). In all the groups with every technique, the treatment was performed on the medial side of the anterior half of the inferior turbinate.

The RFA treatment was carried out with a radiofrequency generator (Sutter RF generator BM-780 II). A “Binner” bipolar needle electrode was inserted into the medial submucosal tissue of the inferior turbinate. The upper and lower parts of the anterior half of the inferior turbinate were treated for 6 seconds at a 10 W output power in 3 areas.

The diode laser treatment was given with a FOX Laser (A.R.C. LASER GmbH, Nuremberg, Germany). The settings were as follows: wavelength of 980 nm, output power of 6 W in continuous wave mode, and a laser delivery by a 600 µm fiber using “contact” mode. Four parallel stripes were made on the mucosa by drawing the fiber from the posterior to the anterior along the medial edge of the anterior half of the inferior turbinate.

In the MAIT treatment, a 2.9 mm-diameter rotatable microdebrider tip (Medtronic Xomed, Jacksonville, Florida, USA) was firmly pushed toward the turbinate bone until it pierced the mucosa of the anterior face of the inferior turbinate. Next, a submucosal pocket was dissected by tunneling the elevator tip in an anterior-to-posterior and superior-to-inferior sweeping motion. Once an adequate pocket had been created, resection of the stromal tissue was carried out by moving the blade back and forth in a sweeping motion, with the system set at 3000 rpm using suction irrigation.

In the placebo procedure, small (2-3 mm in diameter) nasal mucosal biopsies were first taken from the anterior medial portions of the inferior turbinates, causing minor bleeding. Next, a radiofrequency tissue ablation device was turned on repeatedly near the patient, but without the needle electrodes of the device touching the patient; the patient could only hear the acoustic tone of the device. During this sound deception, a suction tube and a nasal endoscope were moved lightly in both sides of the nose for a couple of minutes in order to convince the patients that they had undergone surgery.

### Follow-up and Evaluation

After the operation, none of the patients were given antihistamines or topical treatment, including nasal steroids and nasal decongestants. It was difficult to control the use of medical treatment all the time during the whole 3-year follow-up. However, the patients were strictly forbidden to use topical treatments and antihistamines 1 week prior to evaluation visits to examine the effect of inferior turbinate procedure on their current state regarding nasal obstruction more reliably.

Patients in the placebo group were excluded from the study after 3 months’ follow-up. They were also given a chance to get a genuine surgical treatment outside the study setting. The results of the 3 months’ follow-up with the placebo group have been reported in previous papers.^14,15^ The follow-up of the operated patients continued for 3 years, and 25 patients in the RFA group, 25 in the MAIT group, and 28 in the diode laser group attended both the control visits. Three patients from both the RFA and the MAIT groups withdrew from the study before the end of the follow-up. One patient in the RFA group and one in the MAIT group withdrew from the study due to poor response. Other 2 patients in both groups withdrew from the study for unknown reason.

All the patients were evaluated prior to surgery and 3 months after the surgery. All clinical examinations were performed by the same examiner (TH), who was also the operator and not blinded to the patients’ group knowing, which patients had undergone a placebo operation or a true turbinate reduction. During the visits, the patients also filled a Visual Analog Scale (VAS) regarding the severity of nasal obstruction.

The patients also filled the Glasgow Health Status Inventory (GHSI) questionnaire (Table 1). The Glasgow Health Status Questionnaires—the GHSI and Glasgow Benefit Inventory (GBI)—are developed by the Medical Research Council’s Institute of Hearing Research in collaboration with Glasgow Royal Infirmary, Scotland.^16^ The GHSI is a state questionnaire measuring the effect of a health problem on the sufferers’ QOL. This all-purpose questionnaire is designed to measure especially otorhinolaryngological conditions and is adapted to a specific disease by replacing the words “health problem” in each question with the appropriate problem. The questionnaire contains 18 questions, and the responses are measured with a 5-point Likert scale. A score of 1 indicates a low health status and a score of 5 a high health status. All raw scores are transformed to a scale from 0 to 100. An increase in the score indicates an improvement in the patient’s health status and QOL.

**Table 1.** The Questions in the Glasgow Health Status Inventory (GHSI) Questionnaire.

1. How often does your *nasal obstruction* affect the things you do?
2. How often does your *nasal obstruction* affect your overall life?
3. Which of the following statements best describes your view of the future (5 options)?
4. As a result of your *nasal obstruction*, how often do you feel embarrassment when with a group of people?
5. Is your self-confidence affected by your *nasal obstruction*?
6. How often does any difficulty with your *nasal obstruction* affect how you deal with company?
7. How much support do you have from your friends?
8. How often do you consult your family doctor for any reason?
9. How often does any difficulty with your *nasal obstruction* affect how confident you are about job opportunities?
10. How often does any difficulty with your *nasal obstruction* make you feel self-conscious?
11. How many people really care about you?
12. If there is a cold or infection going around, how often do you usually catch it?
13. How often do you have to take medicine for any reason?
14. Does any difficulty with your *nasal obstruction* affect the way you feel about yourself?
15. How much support do you have from your family?
16. How often are you inconvenienced by your *nasal obstruction*?
17. How often do you participate in social activities?
18. How often do you feel inclined to withdraw from social situations?

Acoustic rhinometry was also performed as an objective examination both before the surgery and during the control visits. V2 to 5 cm value before the decongestant in acoustic rhinometry was used as a parameter for objective examination. It represents well the operated anterior half of the inferior turbinate and describes the possible nasal cavity volume changes due to the operation at that region. All the measurements were carried out at constant temperature and relative air humidity conditions by the same experienced operator to ensure high reproducibility. The background noise level was kept below 60 dB, and the patients also rested and acclimatized 15 to 30 minutes before measurements.

### Statistical Analysis

IBM SPSS Statistics 23.0 was used for the statistical analyses. All non-parametric data were statistically processed using the Wilcoxon signed-rank test and the Kruskal–Wallis test. In the cases with parametric data, the paired samples *t*-test and one-way ANOVA were used.

## Results

Patient characteristics are described in Table 2. Compared to the preoperative values, all 3 techniques improved the VAS score for the severity of nasal obstruction both at 3 months and 3 years (*P* < .001 with all techniques), and there were no significant differences in the VAS score changes between the groups (Table 3).

**Table 2.** Patients’ Characteristics in the Treatment Groups (N = 78).

	RFA	Diode laser	MAIT
Number of patients	25	28	25
Age mean (95% CI)	45.3 (40.1-50.4)	42.5 (37.3-47.7)	46.2 (41.0-51.4)
Sex			
Male, no (%)	12 (48)	16 (57)	16 (64)
Female, no (%)	13 (52)	12 (43)	9 (36)
Allergic sensitization			
Yes, no (%)	10 (40)	12 (43)	10 (40)
No, no (%)	15 (60)	16 (57)	15 (60)

**Table 3.** Changes in the VAS Scores for the Severity of Nasal Obstruction in the Treatment Groups During the Follow-Up.

	RFA (N = 25)	Diode laser (N = 28)	MAIT (N = 25)	*P*-value^a^
Severity of obstruction, median (IQR)				
Preoperative	7.0 (6.0 to 8.3)	8.0 (7.0 to 8.0)	8.0 (7.0 to 9.0)	
3 months	2.0 (1.0 to 3.5)	3.0 (1.6 to 4.0)	3.0 (1.8 to 4.0)	
Change compared to preoperative				
Median (IQR)	−5.0 (−7.0 to −3.0)	−5.0 (−6.0 to −3.0)	−6.0 (−6.3 to −3.0)	NS
Mean (95% CI)	−4.6 (−5.6 to −3.7)	−4.7 (−5.4 to −4.0)	−4.9 (−5.8 to −4.0)	
*P*-value^b^	<.001*	<.001*	<.001*	
3 years	3.0 (1.0 to 7.0)	2.5 (1.5 to 4.8)	3.0 (2.0 to 6.0)	
Change compared to preoperative				
Median (IQR)	−5.0 (−5.5 to −1.5)	−4.3 (−6.4 to −4.0)	−5.0 (−6.0 to −2.0)	NS
Mean (95% CI)	−3.4 (−4.8 to −2.0)	−4.4 (−5.6 to −3.2)	−4.3 (−5.4 to −3.3)	
*P*-value^b^	<.001*	<.001*	<.001*	

*Note.* The distribution of the data is non-parametric, hence medians and interquartile ranges (IQR: Q25-Q75) are used in statistical testing.

Abbreviation: NS, not significant.

a Kruskal–Wallis test.

b Wilcoxon signed-rank test.

* Statistically significant.

All 3 examined techniques improved the GHSI total score compared to the preoperative values both at 3 months and 3 years (*P* < .001 with all techniques), and there were no significant differences in the GHSI total score changes between the groups (Table 4).

**Table 4.** Changes in the Glasgow Health Status Inventory (GHSI) Total Scores in the Treatment Groups During the Follow-Up.

	RFA (N = 25)	Diode laser (N = 28)	MAIT (N = 25)	*P*-value^a^
GHSI total score				
Preoperative, mean (95% CI)	55.7 (50.9-60.5)	57.1 (54.3-60.0)	53.5 (48.0-59.0)	
3 months, mean (95% CI)	68.7 (63.8-73.6)	67.4 (62.6-72.1)	68.3 (63.5-73.0)	
Change compared to preoperative, mean (95% CI)	13.0 (8.9-17.1)	10.2 (6.2-14.3)	14.8 (10.2-19.4)	NS
*P*-value^b^	<.001*	<.001*	<.001*	
3 years, mean (95% CI)	70.1 (64.1-76.0)	70.5 (65.5-75.5)	67.7 (62.2-73.2)	
Change compared to preoperative, mean (95% CI)	14.4 (8.6-20.1)	13.4 (9.3-17.5)	14.3 (8.9-19.6)	NS
*P*-value^b^	<.001*	<.001*	<.001*	

Abbreviation: NS, not significant.

a One-way ANOVA.

b Paired samples *t*-test.

* Statistically significant.

Compared to the preoperative values, the RFA (*P* = .001), MAIT (*P* = .001), and diode laser (*P* = .008) techniques all increased the V2-5 cm values at 3 months, and there were no significant differences in the V2-5 cm value changes between the groups. At 3 years, compared to the preoperative values, the MAIT (*P* = .005) and diode laser (*P* < .001) techniques, but not RFA (*P* = .06), increased the V2 to 5 cm values (Table 5).

**Table 5.** Change in V2-5 cm Acoustic Rhinometry Values (1 Side of the Nasal Cavity).

	RFA	Diode laser	MAIT	*P*-value^a^
V2-5 cm (cm^3^)				
Preoperative, median (IQR)	4.54 (3.16 to 5.71)	3.47 (2.71 to 4.36)	3.58 (2.55 to 4.64)	
3 months, median (IQR)	5.81 (4.29 to 7.13)	4.37 (3.56 to 5.60)	4.24 (3.40 to 5.38)	
Change compared to preoperative				
Median (IQR)	1.00 (−0.47 to 2.55)	0.84 (−0.41 to 1.66)	0.58 (−0.31 to 1.38)	NS
Mean (95% CI)	1.33 (0.56 to 2.10)	0.85 (0.43 to 1.26)	0.70 (0.19 to 1.21)	
*P*-value^b^	.001*	.001*	.008*	
3 years, median (IQR)	5.01 (4.04 to 5.87)	4.47 (3.72 to 5.43)	4.45 (3.36 to 6.36)	
Change compared to preoperative				
Median (IQR)	0.53 (−0.80 to 2.06)	1.08 (0.15 to 1.68)	1.18 (−0.37 to 2.60)	NS
Mean (95% CI)	0.60 (0.01 to 1.20)	1.07 (0.55 to 1.59)	1.18 (0.45 to 1.92)	
*P*-value^b^	.06	<.001*	.005*	

*Note.* The distribution of the data is non-parametric, hence medians and interquartile ranges (IQR: Q25-Q75) are used in statistical testing.

Abbreviation: NS, not significant.

a Kruskal–Wallis test.

b Wilcoxon signed-rank test.

* Statistically significant.

## Discussion

Classical submucosal resection, where the vertical part of the conchal bone and a wedge of soft tissue attached to the lateral and inferior aspects are resected,^1^ has been shown to result in a good long-term degree of nasal patency in 5- and 6-year follow-ups.^17,18^ MAIT can be considered a powered subtype of the submucosal resection technique. In MAIT, the debridement of the submucosal tissue from the inferior turbinate is performed intraturbinally in the medial submucosal plane with a microdebrider blade.^1^ Yanez and Mora treated 350 non-allergic patients with MAIT in a study with a 10-year follow-up. At 10 years postoperatively, 91% of patients were completely symptom-free, and rhinomanometry also revealed a long-term improvement.^9^

Since described by Li et al^19^ RFA has probably become the most used inferior turbinate reduction technique. However, the evidence of the long-term effect of RFA is controversial. Liu et al^20^ found an improvement in VAS and rhinomanometry at 6 months that was only sustained to 1 year, with a gradual return to baseline from 2 to 3 years postoperatively. There are other studies, however, where the clinical benefit of the procedure has persisted for up to 5 years.^10,21^

Since the early 1980s, various types of lasers have also been used for the reduction of enlarged inferior turbinates.^22^ In recent years, diode laser with the used wavelengths of 940, 980, and 1470 nm has gained increasing popularity for its ease of use under local anesthesia.^7,23,24^ In previous studies, the diode laser has been described as providing significant improvement in nasal airflow subjectively and also in objective measurements in 1-year,^7,22^ 2-year,^24^ and 3-year follow-ups.^8^

There are few previous studies that have compared the long-term effect of the MAIT, RFA, and diode laser techniques. Liu et al prospectively treated 120 patients with MAIT or RFA. MAIT resulted in an improvement of the VAS score for nasal obstruction and rhinomanometry results at 6 months with a sustained improvement up to 3 years. RFA also improved the results at 6 months, but the response was sustained only to 1 year, with a return to baseline from 2 to 3 years.^20^ Veit et al compared anterior turbinoplasty (submucosal resection), RFA, and diode laser—each in combination with septoplasty—in a prospective single-blinded randomized trial with 60 patients. A significant subjective improvement in nasal breathing and nasal volume was found at 3 months, 1 year, and 2 years in the anterior turbinoplasty and RFA groups. In the diode laser group, a significant improvement in nasal breathing was observed only at 3 months. Furthermore, the diode laser group also failed to reach significant improvement in nasal volume at all follow-up visits.^25^ To date, no studies have compared the MAIT, RFA, and diode techniques together.

In the present study, all 3 examined techniques improved the VAS score for the severity of nasal obstruction at 3 months, and as concluded in our previous paper on the three-month follow-up, provided a statistically significant additional reduction in the severity of nasal obstruction compared to the placebo procedure.^14^ Compared to the preoperative values, the statistically significant improvement was sustained for the whole follow-up period of 3 years with all 3 techniques. The results of the anterior nasal cavity volume (V2-5 cm) in acoustic rhinometry, which we used as a parameter for objective examination, also improved with all 3 techniques at 3 months. However, compared to the preoperative values, the statistically significant improvement was sustained in the longer follow-up period of 3 years only in the MAIT and diode laser groups, but not in the RFA group.

Regarding the objective examination results of the RFA technique, our findings resemble the results of Liu et al^20^ at some point. Although the results of the V2 to 5 cm value in the RFA group did not return completely to the preoperative baseline, a decrease in the treatment response can be seen in the longer follow-up. RFA is an electrosurgical procedure that creates a submucosal lesion, inducing tissue volume reduction. RFA causes a controlled tissue lesion with a temperature of less than 85°C, which is far less than the temperature of around 800°C brought about by electrocautery and lasers.^1^ The lesser tissue damage caused by the lower heat in the first place compared to potentially greater damages caused by the higher temperature in diode laser procedures or direct surgical submucosal resection in MAIT procedures is one possible explanation for the decreased V2 to 5 cm value only in the RFA group. However, the weakening of the anterior nasal cavity volume response did not cause a significant decrease in the subjective long-term response to the RFA procedure. The finding of the sustained long-term improvement of the subjective response as well as the fact that the technique is very easy to use under local anesthesia in the office setting still justifies its use in the treatment of chronic nasal obstruction.

There are few previous studies evaluating the long-term effect of the MAIT, RFA, and diode laser procedures on the patients’ quality of life. RFA has been found to improve patients’ QOL significantly in 1-year and 2-year follow-ups and MAIT in 1-year follow-ups.^26-28^ No studies have compared the MAIT, RFA, and diode techniques together.

In the present study, the effect of the procedures on the QOL was assessed with the GHSI, which has been shown to be a valid, reliable, and responsive QOL measurement instrument in chronic nasal obstruction.^29^ All the 3 examined techniques improved the GHSI total score at 3 months, indicating improvement in the QOL, and the statistically significant improvement was sustained for the whole follow-up period of 3 years with all 3 techniques.

All clinical evaluations and surgical procedures in this study were performed by the same physician, who was not blinded to the patient’s groups knowing, which patients had undergone a placebo operation or a true turbinate reduction. Unblinding of the examining physician could cause unintentional bias by causing misinterpretation of data thus spoiling the reliable comparison of the treatment groups. However, the examination parameters used in this study were not based on the subjective evaluation of the examining physician. The objective examination of the patients, in turn, was carried out with acoustic rhinometry, which was preferred over other alternative means, such as deidentified videos with a blinded observer, which are based on the subjective assessment of the examiner. After the operation the patients were advised not to use antihistamines or topical treatments. However, during the long follow-up period, it is very difficult to enforce the compliance of avoiding the medical treatment all the time. Therefore, the possible lack of compliance of some patients may have affected the results to some extent.

The results of the study clearly indicate that all 3 techniques are efficient in improving the patients’ subjective symptom of chronic nasal obstruction and QOL, and the response is sustained for up to 3 years. We also found a weakening in the objective treatment response to RFA in the longer follow-up. However, technical difficulties and environmental conditions may always have some influence on the volume values in acoustic rhinometry, which must be considered when assessing the result. A larger study population in the treatment groups could also have made the result more reliable. Regarding the RFA finding, further studies with long-term follow-ups are needed. In addition, there is no determined minimal clinically important difference value for the GHSI questionnaire. Although statistically significant GHSI total score changes of the same magnitude as in the present study can be found in the recent rhinological literature,^30^ the clinical meaning of the GHSI change finding for the patient is not entirely confirmed.

## Conclusion

The RFA, MAIT, and diode laser all improved the patients’ subjective sensation of the severity of nasal obstruction and the QOL significantly. The response was sustained in the follow-up period of 3 years with all 3 techniques. Weakening in the objective treatment response to RFA was found in the longer follow-up, but that did not cause a weakening of the patients’ subjective treatment responses. These findings support the conclusion that the examined techniques are effective in the treatment of chronic nasal obstruction and give the patients long-term help.
